# The effect of metabolic surgery on type 1 diabetes: meta-analysis

**DOI:** 10.20945/2359-3997000000021

**Published:** 2018-03-23

**Authors:** Abdulzahra Hussain

**Affiliations:** 1 Doncaster and Bassetlaw Teaching Hospitals Doncaster UK Doncaster and Bassetlaw Teaching Hospitals, DN2 5LT, Doncaster, UK

**Keywords:** Bariatric surgery, metabolic surgery, type 1 diabetes mellitus, type 2 diabetes mellitus, HBA1c, insulin, body mass index

## Abstract

**Objective:**

Metabolic and bariatric surgery has a definite role in the management of obese patients with type 2 diabetes mellitus (T2DM). There is also evidence of such surgery improving the health of type 1 diabetic (T1DM) patients. The aim of this paper is to explore the effect of metabolic and bariatric surgery on T1DM.

**Materials and methods:**

A comprehensive search of PubMed and Google Scholar was performed to identify relevant papers reporting metabolic and bariatric surgery effects on T1DM. A statistical analysis is applied after data synthesis. A forest plot and Pearson correlation are then calculated.

**Results:**

Of the 567 papers that were identified, 558 articles did not fulfill the inclusion criteria and were therefore excluded. Nine studies involving 78 patients were selected for this metaanalysis. There was improvement in HBA1c (p value = 0.40), insulin dose (p value = 0.0001) and BMI (p value = 0.00001) after surgery. However, improvement in the HBA1c did not reach statistical significance. There was a weak correlation between postoperative insulin dose and BMI change after surgery (r = -0.177). There was a negligible correlation between HBA1c and BMI change after operations (r = -0.01).

**Conclusion:**

Current metabolic/bariatric surgery is improving T1DM in obese and morbidly obese patients. This is not exclusively related to excess weight loss (EWL) as previously thought. Therefore, there is a role for other factors, which are potential players to reproduce the same effect in nonobese T1DM patients.

## INTRODUCTION

Gastrointestinal surgery has excellent but variable outcomes on the glycemic control of diabetic patients depending on the type of surgery. The first report of gastrointestinal surgery ameliorating T2DM was reported by Friedman and cols. in 1955 (1), though its effect on T1DM was not recognized until recently when Czupryniak and cols. reported the first observation of T1DM improvement in a severely obese patient who underwent gastric bypass in 2004 (2). Hussain and cols. suggested the potential benefit of bariatric surgery for T1DM in 2009 (3), followed by Czupryniak and cols.'s case studies involving three patients in 2010 (4). Since then, several observational studies have reported changes in insulin requirement, HBA1c and BMI following different types of bariatric/metabolic surgery.

American Diabetic Association (ADA) and National Institute for Health and Care Excellence (NICE) guidelines have restricted the diagnosis of T1DM to situations in which the body does not produce insulin (5) or the destruction of insulin-producing beta cells in the pancreatic islets of Langerhans causes absolute insulin deficiency (6). This clear definition should avoid confusion in reporting insulin-dependent diabetic patients after bariatric/metabolic surgery, which could fall under either T1DM (when there is no insulin production) or T2DM (when insulin is produced but is not a sufficient amount for body requirements, or there is insulin resistance).

The metabolic effect of bariatric/metabolic surgery on T1DM has elicited significant interest because of the already-proven benefits on T2DM and the potential production of similar results for T1DM, which forms 10% of diabetic load (7–10). As there is no insulin production by pancreatic beta cells, the mechanisms of improving T1DM following metabolic/bariatric surgery are expected to be related to body mass index (BMI) change, reduction of insulin resistance, satiety/dietary change and possible neuroendocrine/hormonal or incretins influence. The aim of this paper is to explore current evidence regarding the effect of metabolic surgery on T1DM.

## MATERIALS AND METHODS

**Figure 1:**
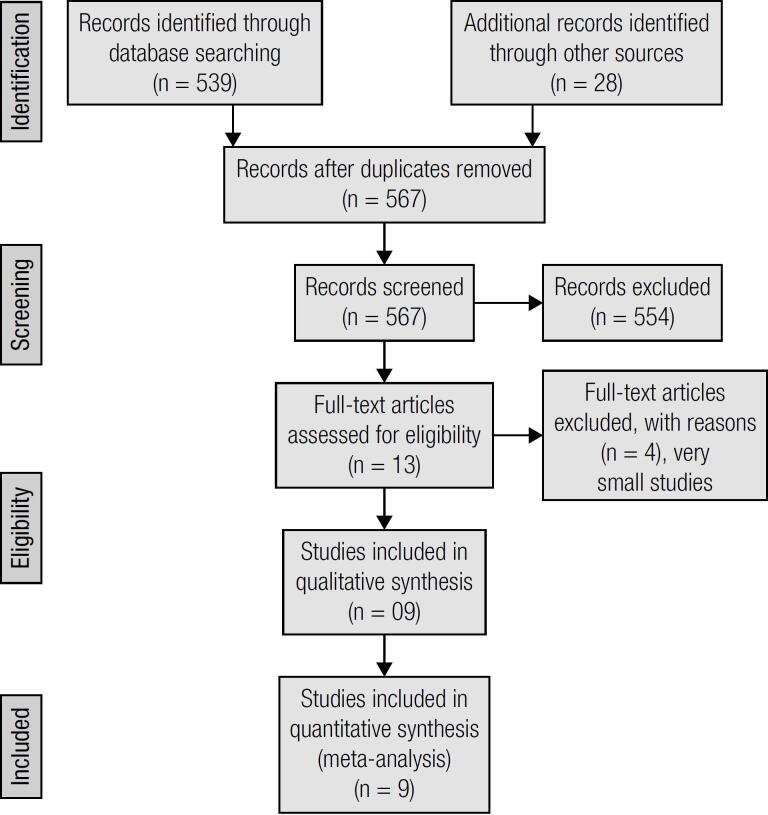
Flow diagram.

Source: Moher D, Liberati A, Tetzlaff J, Altman DG; The PRISMA Group (2009). Preferred Reporting Items for Systemic Reviews and Meta-Analyses: The PRISMA Statement. PLoS Med. 2009;6(7):e1000097.

The protocol of Preferred Reporting Items for Systematic Review and Meta-Analysis (PRISMA) is used (Figure 1). Inclusion and exclusion criteria: All English literature reporting bariatric and metabolic surgery on T1DM patients (hyperglycemia, C-peptide negative and anti-glutamic acid carboxylase [GAC] antibodies positive) were included. The studies that reported less than three patients were excluded.

### Quality of the data

An assessment of the studies according to the Newcastle-Ottawa scoring system was performed (Table 1).

**Table 1:** Newcastle-Ottawa assessment of the quality of data

Studies	Selection	Comparability	Outcome
Blanco and cols. (2014)	****	*	**
Brethauer and cols. (2014)	***		**
Czupryniak and cols. (2010)	***		**
Maraka and cols. (2015)	****	*	**
Middelbeek and cols. (2015)	***		**
Raab and cols. (2013)	***		**
Robert and cols. (2015)	****	*	**
Lannoo and cols. (2014)	****		**
Mendez and cols. (2010)	***		**

The data were reviewed and entered into the fields of Microsoft Excel. The data consisted of the following: sample size, authors, year of publication, HBA1c, insulin dose, BMI before and after the surgery and follow-up duration. All the studies were observational research, and no clinical trials were to have been performed for the studies to be included. Three of them were comparative protocols that included a T2DM arm. Only T1DM data were selected. Some of the studies lacked the calculation of standard deviation (SD), which is an important requirement for conducting the forest plot calculation. The SD of each study was calculated from the number of subjects, the largest and smallest value and the 95% confidence interval of 3.92. Some of the studies were lacking a mean of the specific data. Therefore, a calculation of the mean was performed. The patients’ follow-ups varied, even within a single study. Some studies provided follow-up at 6 months, 1 year, 2 years, 3 years, 4 years or 5 years. The included data were taken from the longest follow-up to give more power to the results. Few studies reported insulin dose per kilogram (kg) of body weight/day, while the majority quoted total units/day. It was not possible to know the total units/day for these few studies (Table 2), so the reported unit/kg/day is used for analysis.

**Table 2:** Main data, the standard deviation is calculated using the formula SD = √n x[X-x)^2^/3.92, n = number of subjects, X = upper limit value, x = lower limit, 3.92 = 95% confidence interval, CI. The values in the table represent the mean of the data plus SD when available. For insulin requirements, some data depicted as IU/kg/day rather than total unit/day

	Year	Age (mean) years	No of subjects	HBA1c before/after surgery	Insulin requirements before/after surgery IU/day (u/k/day)	BMI kg/m^2^ before/after surgery	Duration of T1DM in years	Follow up (month = m)
Czupryniak and cols.	2010	22 ± 6	3	10.13 ± 1.32/09.00 ± 0.73	94 ± 6.3/47.6 ± 6.42	42.2 ± 2.39/33.5 ± 2.65	11.60	5-8 years
Raab and cols.	2013	43	6	8.18 ± 2.17/6.95 ± 1.4	104.16 ± 11.59/36.75 ± 7.72	41.75 ± 5.89/27.82 ± 2.37	17.16	1 year
Robert and cols.	2015	39.2 ± 5.3	10	7.5 ± 1.9/7.1 ± 0.9	1.09 ± 0.7/0.44 ± 0.24	46.9 ± 6.3/30.34 ± 6.3	23.1 ± 11.8	4.5 years
Maraka and cols.	2015	50.6 ± 8.9	7	8.2 ± 1.6/7.8 ± 0.9	75.15 ± 6.4/55.45 ± 5.17	44.3 ± 8.0/31.2 ± 9.9	20.6 ± 11.4	2 years
Blanco and cols.	2014	38.2 ± 13.3	7	8.3 ± 1.2-8.2 ± 0.9	0.61 ± 0.17/0.62 ± 0.12	39.4 ± 2.2/27.3 ± 2.2	27.3 ± 2.2	2 years
Middelbeek and cols.	2015	39.6 ± 8.4	10	8.1 ± 1.3/9.8 ± 1.9	53.0 ± 29.7/31.1 ± 22.8	43.5 ± 7.5/33.8 ± 7.5	24.6 ± 10.1	5 years
Brethauer and cols.	2014	45.6 ± 10.9	10	10 ± 1.6/8.9 ± 1.1	0.74 ± 0.32/0.40 ± 0	41.6 ± 3.9/30.5 ± 5.9	22	36.8 ± 32.3 (m)
Lannoo and cols.	2014	40.3 ± 8.5	22	8.4 ± 2.34/8.2 ± 2.11	92.5 ± 13.81/48.0 ± 11.36	39.7 ± 5.34/31.4 ± 2.86	25.3 ± 8.9	14.3 ± 10.1/37.8 ± 29.7
Mendez and cols.	2010	42.3	3	7.96 ± 0.67/8.03 ± 0.99	99.23 ± 9.36/42.9 ± 3.10	45.9 ± 3.11/29.4 ± 1.77	25	1 year

### Data synthesis

**Figure 2:**
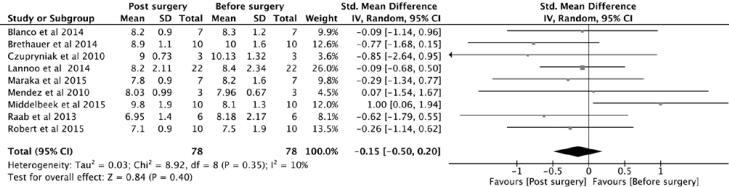
Forest plot, HBA1c.

**Figure 3:**
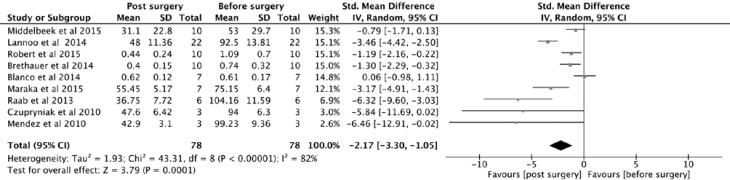
Forest plot, insulin.

**Figure 4:**
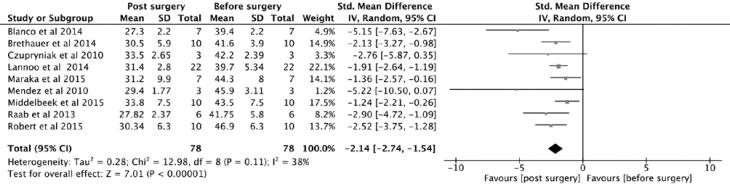
Forest plot, body mass index change after surgery.

Data synthesis was completed and is shown in Table 2. Further calculation of change in the mean of three variables is depicted in Table 3. The forest plot was performed for HBA1c, Insulin dose and BMI changes after surgery (Figures 2, 3 and 4). Pearson correlation statistics were applied, and the results are shown in Table 4.

**Table 3:** Mean change in 3 variables BMI, HBA1c and insulin requirement, mean duration of T1DM (years) and age (years) are fixed

HBA1c	Insulin requirement	Duration of T1DM	BMI	AGE
1.13	46.40	11.60	08.70	22.0
1.23	67.41	17.16	13.95	43.0
0.40	00.65	23.10	16.56	39.2
0.40	19.70	20.60	13.10	50.6
0.10	00.01	27.30	12.10	38.2
-1.70	21.90	24.60	09.70	39.6
0.10	00.34	22.00	11.10	45.6
1.10	56.33	25.30	08.30	40.3
-0.07	44.50	25.00	16.50	42.3

**Table 4:** Pearson correlation for 2 constant (age and duration of T1DM) and 3 variable post operative parameters (insulin requirements, HBA1c change and BMI change) combination. Significant moderate correlation between any two parameters is depicted in grey color (0.-0.39 = weak, 0.4-0.59 moderate, 0.6-0.79 = strong, 0.8-1.0 = very strong, Evans guide 1996)

	Age	Duration of T1DM	Insulin requirement	HBA1c	BMI change after surgery
Age	-----	0.509	-0.208	-0.220	0.405
Duration of T1DM	0.509	------	-0.418	-0.513	0.204
Insulin requirement	-0.208	-0.418	------	0.475	-0.177
HBA1c	-0.210	-0.513	0.475	-----	-0.010
BMI	0.405	0.204	-0.177	-0.010	------

### Statistical analysis

Review Manager software was used (Review Manager 5.3, version: 5.3.5). A 95% confidence interval (CI) and p < 0.05 limit was taken as significant. An odd ratio (OR), variance, standard errors and deviations were applied, and a forest plot for depicting the final results of each parameter was constructed. Different parameters such as age, insulin requirements, HBA1c, BMI and duration of diabetes were included in the final analysis. Further analysis was conducted to show the relations and degree of correlation between these 5 parameters. The correlation tests were performed using Excel (Microsoft 365, 2015) and also R software from the R Foundation for Statistical Computing (http://www.Rproject.org). The test of normality was conducted for all data using Jarque-Bera and Anderson-Darling normality tests. The correlation coefficient (r) was calculated using the following equation: *R Calculation*, r = ∑((X - M_y_)(Y - M_x_)) / √((SS_x_)(SS_y_)). Data synthesis was performed, and statistical analysis was conducted. The SD was lacking in some studies, and it was calculated using SD = √Nx (X-x)/3.92 and a confidence interval of 95% (http://handbook.cochrane.org).

## RESULTS

### Type of bariatric/metabolic procedure

The included patients underwent laparoscopic adjustable gastric band (LAGB), 2 patients (2.5%); vertical sleeve gastrectomy (VSG), 11 patients (14%); laparoscopic Roux-en Y gastric bypass (LRYGB), 52 patients (67%); bilio-pancreatic diversion (BPD) 7 patients (9%); or bilio-pancreatic diversion plus duodenal switch (BPD-DS), 3 patients (3.8%). Except gastric bypass, all other operations included a small number of patients who fell short in terms of providing statistical power, and no subgroup analysis was conducted. Therefore, the outcomes were those of the 5 procedures.

### HBA1c

HBA1c showed improvement after surgery; however, this improvement did not reach statistical significance, as the p value was 0.40 (Figure 2). HBA1c was moderately correlated with the duration of T1DM (r = -0.513) and insulin requirement (r = 0.475). HBA1c showed little or no correlation with BMI (r = 0.01) but a stronger correlation with age (r = -022).

### Insulin requirement

Insulin requirement was significantly reduced after surgery, with p value = 0.00001 (Figure 3). It was moderately correlated with HBA1c, as expected (r = 0.475), and with duration of T1DM (r = -0.418). More importantly, it was weakly correlated with BMI change after surgery (r = -.0177) and also with age (r = -0.208) (Figures 2 and 4).

### Duration of the diabetes

The mean duration of T1DM was 9.6 to 34.9 years, and it was correlated with age (r = 0.509). Duration of T1DM was moderately correlated with post-surgery insulin requirements and HBA1c and weakly correlated with BMI (r = -0.418, -0.513, 0.204, respectively) (Table 4).

### Excess weight loss

All studies showed an acceptable amount of weight loss ranging from 8.3 to 16.56 kilograms/m^2^. BMI change after surgery was weakly correlated with duration of diabetes, HBA1c and insulin requirement; however, it had significant correlation with age (r = 0.405) (Table 4).

### Age group

The age group ranged from 16-65 years with mean of 40.16 years. There was a weak negative correlation with postoperative insulin dose and HBA1c (r = -0.205, -0.21, respectively). Age group was moderately correlated with duration of T1DM and BMI (r = 0.509, 0.405, respectively).

## DISCUSSION

HBA1c and postoperative insulin dose are important parameters to assess glycemic control in T1DM patients following metabolic surgery. All but 2 studies (Middelbeek and cols. 2015 and Mendez and cols. 2010) showed a variable degree of improvement in HBA1c, although it did not reach statistical significance (4,11–18) (Figure 2). However, the insulin dose is significantly reduced following surgery, and it is moderately correlated with duration of T1DM and HBA1c level. This meta-analysis showed a weak correlation among postoperative HBA1c, insulin requirement and postoperative BMI loss. Therefore, the improvement in the HBA1c and insulin dose shortly after surgery is not entirely related to weight loss. The largest study of Lannoo and cols. (11) concluded that the insulin-sparing effect is probably related to insulin sensitivity following weight loss. However, according to this meta-analysis, the insulin-sparing effect is only weakly correlated with weight loss. We have to look beyond the anatomical configurations and physical effects of these procedures and explore the complex metabolic pathway of glucose homeostasis.

Incretins play a role in glucose regulation by reducing glucagon and food intake and increasing satiety (19). A recent review study highlighted the established roles of gut hormones in regard to diabetes (20). Metabolic and bariatric surgery may possibly produce insulin dose reduction and improve HBA1c through an incretinsrelated mechanism, but this does not exclude other potential factors that control glucose metabolism (21). The change in the nutrient flux could affect the balance of gut hormones (including hypothetical anti-incretins), and the change in hormone milieu might be responsible for changes in insulin sensitivity (22).

In their study, Kempf and cols. showed a rapid decrease of insulin requirement and an improvement in HBA1c in T2DM as a result of meal modification even before substantial weight loss occurred (23). Glycogenolysis, gluconeogenesis, carbohydrate intake and the modified response of the metabolism to postmetabolic/bariatric surgery in regard to anatomical, neuroendocrine and satiety changes are the primary cofactors for net glucose production. The interaction of these complex mechanisms produces the final blood glucose level, whether it is normal, hypoglycemic or hyperglycemic.

Following procedures such as gastric bypass, some T2DM patients develop hypoglycemia. The incidence of symptomatic hypoglycemia is less than 1% (24). However, asymptomatic post-bariatric/metabolic surgery hypoglycemia could reach 30% (25). In these patients, we possibly overdoing metabolic correction. On the other hand, in T1DM, the beta-cell influence on glucose homeostasis is absent, and therefore the entire set of regulatory mechanisms consists of the complex interactions among the gastrointestinal tract (GIT), liver, brain, adipose tissue, blood cells, muscles and kidneys. This results in the regulation of glucose entry into the circulation being influenced by additional factors such as hormones, the sympathetic nervous system and substrates (i.e., free fatty acid concentrations and availability of gluconeogenic precursors) (26).

The question regarding how to adjust the anatomical change of the gastrointestinal tract as a result of metabolic surgery and the inherent complex interactions among these systems to optimize the glucose level in T1DM is very difficult based on the current level of knowledge surrounding metabolic/bariatric surgery. Every single centimeter of the GIT is a complex unit, and any change caused by metabolic and bariatric surgery will result in a comparative impact to the glucose homeostasis. Such changes will never result in total glycemic control, and the patient will still need an insulin replacement, regardless of the preoperative insulin dose. Currently, we do not entirely know what each part of the GIT produces (except some factors such as incretins, PYY hormones and ghrelins), and we are still a far way from being in a position to perfect metabolic/bariatric surgery to produce the maximum benefit for T1DM patients. The interaction between diet and GIT has been a very important focus of interest. A very low calorie diet (VLCD) was found to produce similar effects to gastric bypass in terms of insulin sensitivity and beta-cell function improvement in T2DM (27). As diabetes is a spectrum of disease, the same effect could therefore be produced in T1DM. To date, no study has examined the potential effect of VLCD on T1DM. Bile acids are recognized effectors on the regulation of glucose and lipid metabolism through the FXR and TGR5 receptors (28). There is evidence that the alteration of bile acids following bariatric surgery improves insulin responsiveness and lowers fasting glucose in animal models (29). A similar effect may be reproduced in humans, and it may be a mechanism through which to explain the effect of metabolic surgery on diabetic patients.

There is no direct link between gut microbiota and improvement in glucose homeostasis following bariatric surgery. Gut microbiota does, however, have a direct effect on weight. An interesting study showed that transferring microbiota from an obese subject to a lean subject resulted in weight gain (30). The hepatocytes orchestrate the regulatory mechanisms of bile acids, microbiota and metabolome to affect glucose and lipid metabolism (31). Future research may prove the existence of a link between microbiota and beta-cell function.

### Limitations of the study

This meta-analysis included 9 studies with some degree of heterogeneity that ranged from 10-82%. The studies are relatively small, with the largest reporting 22 patients. The patient follow-ups are different, as some studies reported 1 year, whereas others reported up to 5 years. The outcomes, especially HBA1c, may be creeping up with longer follow-up, as shown by Middelbeek and cols.'s 2015 study (12), and the current conclusion regarding HBA1c is represented by the mean followups in these studies. Some studies included women only (like Middlbeek and cols. 2015), whereas others reported women as the majority. This would raise a question regarding the actual representation of the obese T1DM population; nevertheless, the studies are shedding a light on such patients who require extra help with glycemic control after having exhausted current nonsurgical methods.

## CONCLUSION

Current metabolic/bariatric surgery is reducing postoperative insulin requirement and marginally improving HBA1c in obese and morbidly obese type 1 diabetic patients. This is not exclusively related to the EWL as previously thought. Therefore, there is a role for other factors, which are potential players to reproduce the same effect in nonobese T1DM patients. Further long-term studies are required to assess the real benefit of metabolic surgery for T1DM patients.
